# Nonlinear plasmonic response in atomically thin metal films

**DOI:** 10.1515/nanoph-2021-0422

**Published:** 2021-10-15

**Authors:** Álvaro Rodríguez Echarri, Joel D. Cox, Fadil Iyikanat, F. Javier García de Abajo

**Affiliations:** ICFO-Institut de Ciencies Fotoniques, The Barcelona Institute of Science and Technology, 08860 Castelldefels, Barcelona, Spain; Center for Nano Optics, University of Southern Denmark, Campusvej 55, DK-5230 Odense M, Denmark; Danish Institute for Advanced Study, University of Southern Denmark, Campusvej 55, DK-5230 Odense M, Denmark; ICREA-Institució Catalana de Recerca i Estudis Avançats, Passeig Lluís Companys 23, 08010 Barcelona, Spain

**Keywords:** atomically thin films, nonlinear optics, nanophotonics, plasmon polaritons, scanning near-field optical microscopy (SNOM)

## Abstract

Nanoscale nonlinear optics is limited by the inherently weak nonlinear response of conventional materials and the small light–matter interaction volumes available in nanostructures. Plasmonic excitations can alleviate these limitations through subwavelength light focusing, boosting optical near fields that drive the nonlinear response, but also suffering from large inelastic losses that are further aggravated by fabrication imperfections. Here, we theoretically explore the enhanced nonlinear response arising from extremely confined plasmon polaritons in few-atom-thick crystalline noble metal films. Our results are based on quantum-mechanical simulations of the nonlinear optical response in atomically thin metal films that incorporate crucial electronic band structure features associated with vertical quantum confinement, electron spill-out, and surface states. We predict an overall enhancement in plasmon-mediated nonlinear optical phenomena with decreasing film thickness, underscoring the importance of surface and electronic structure in the response of ultrathin metal films.

## 1 Introduction

The search for materials that exhibit a large nonlinear optical response at reduced light intensity thresholds has been a prominent theme in the optical sciences ever since the laser was introduced [1–6]. Nonlinear optical phenomena are now routinely accessed by phase-matching high-power laser light in macroscopic bulk crystals [7] or atomic gases [8]. The frontier of nano optics, where the inherently small light–matter interaction volumes of nanostructured materials limit the accumulation of an appreciable nonlinear response, presents a considerably more challenging arena in which to control light by light. The situation can be partly alleviated through electronic band structure engineering [9], boosting the intrinsic nonlinear response of a material, or by exploiting the near-field enhancement supplied by subwavelength optical resonances [10]. These strategies can be applied in the mesoscopic regime using low-dimensional materials, which constitute a configurable platform for actuating nonlinear optical effects on the nanoscale [11, 12].

Ultrathin metal films with thickness down to the few-atomic-layer level can support extremely confined plasmons [13–18] that hold high potential for disruptive optoelectronics applications in the visible and near-infrared regimes [19, 20], thus complementing similar capabilities developed in the context of graphene plasmonics [21–24], which unfortunately is currently limited to the mid-infrared range. A recent experiment reveals that plasmons possess long lifetimes in few-atom-thick crystalline samples [18]. Additionally, in analogy to graphene plasmons [12], ultrathin metal films and their heterostructures have been predicted [25] and demonstrated [26, 27] to offer an enhanced nonlinear optical response due to their quantum-confined electronic states.

Plasmonic near-field enhancement offers a tantalizing route towards nanoscale nonlinear optics, motivating experimental and theoretical research in *nonlinear plasmonics* [4]. In this context, patterned metallic nanostructures are commonly utilized for their ability to in- and out-couple *localized* plasmons and far-field radiation [28, 29]. In contrast, propagating surface plasmon polaritons (SPPs), characterized in ultrathin films by extremely compressed wavelengths compared to those of freely propagating photons with the same frequency, must be launched by evanescent fields to satisfy energy-momentum conservation [30], and hence, the nonlinear optical response associated with SPPs is less commonly probed in experiments [31], despite the appeal of nonlocal control over nonlinear interactions of propagating SPPs. Furthermore, many of the exciting properties of plasmons in ultrathin films that emerge from vertical electron confinement also rely on the preservation of 2D translational symmetry, which becomes crucial for high-quality crystalline samples to exhibit lower losses than their amorphous counterparts [18, 32].

The first experimental venture in nonlinear plasmonics led to the observation of enhanced second harmonic generation (SHG) from a ≈56 nm silver film in reflection [33], in agreement with the Fresnel coefficients constructed from tabulated linear and nonlinear response functions of silver available at that time. SHG at a metal surface, which provides the requisite breaking of inversion symmetry in a centrosymmetric medium [34, 35], demands a more sophisticated theoretical model to account for nonlocal effects, which are well described in metals through the random-phase approximation (RPA) within the linear regime [36] and in the second-order nonlinear response through an extension of the RPA [37]. In particular, the RPA prescription captures quantum-well states in the optical response of metal films, which have been demonstrated to play an important role in nonlinear phenomena when the film is comprised of only several atomic layers [38–41]. Obviously, monolayer crystals, and more precisely systems that exhibit nonparabolic electronic band structure, display strong nonlocal nonlinear effects, as revealed in recent theoretical [12] and experimental [42] studies on graphene. Alternatively, two-dimensional transition metal dichalcogeneides, with unique crystal structures that may exhibit centrosymmetry or noncentrosymmetry depending on the number of layers, have demonstrated relatively large nonlinear yields [43–48]. In parallel efforts, the nonlinear optical properties of polycrystalline ultrathin noble metal films have also been measured [26, 49, 50] and shown to hold great potential for applications.

Here, we theoretically explore the nonlinear optical response associated with plasmons in few-atom-thick crystalline noble metal films, an emerging high-quality material platform for nanophotonics [18]. We introduce rigorous theory based on a quantum–mechanical description of the SPP-mediated nonlinear optical response in crystalline metal thin films, and in the spirit of motivating experimental investigations, we simulate the signal produced by evanescent fields encountered in near-field characterization techniques such as the Kretschmann configuration, scanning near-field optical microscopy (SNOM), or optical gratings. Focusing in particular on ultrathin silver films with (111) crystallographic orientation, our calculations reveal a remarkable improvement in the nonlinear optical yield of second- and third-order processes with decreasing film thickness, emphasizing the role of surface and quantum finite-size effects in the nonlinear plasmonic response of ultrathin metal films. In addition, low-energy features associated with Shockley surface states supported by the (111) crystalline film are predicted to emerge in the nonlinear optical response. We expect that our results will inspire future explorations in nonlinear nano optics using crystalline noble metal films that are currently available in experiment [18].

## 2 Results and discussion

**Figure 1:**
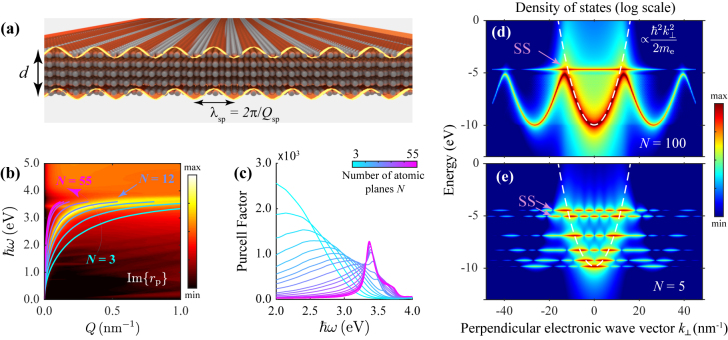
Plasmonic near-field and electronic structure of crystalline thin films. (a) Illustration of an SPP excited in an ultrathin silver film comprised of *N* stacked (111) atomic layers with period *a*_*s*_, such that *d* = *Na*_*s*_ is the effective film thickness. (b) Contour plot (log scale) showing the in-plane-wave-vector and photon-energy dependence of the imaginary part of the loss function Im{*r*_p_} for an Ag(111) film consisting of *N* = 12 atomic layers. We superimpose plasmon dispersion curves signaled by maxima in the loss function Im{*r*_p_} for films of thickness indicated by the color scale in panel (c). (c) Spectral dependence of the Purcell factor for films of thickness indicated by the color scale. (d)–(e) Electronic density of states obtained by Fourier-transforming the out-of-plane electronic wave functions *φ*_*j*_(*z*) of occupied quantum states to out-of-plane wave-vector space for films consisting of (d) *N* = 100 and (e) *N* = 5 Ag(111) atomic layers. A Lorentzian spectral broadening of 21 meV FWHM is introduced. The superimposed dashed white curves indicate the in-plane parabolic dispersion of the first quantum-well state *j* = 1. Surface states, characteristic of the (111) crystallographic orientation, are highlighted by purple arrows.

Within the framework of classical electromagnetism, a metal film of thickness *d* characterized by a Drude-like permittivity predicts a frequency-dependent in-plane SPP wave vector $Q_{\text{sp}}\left(\omega \right)=\left(2/d\right)\;\coth^{-1}\left[\left(\omega_{\text{p}}^{2}/\omega^{2}-\epsilon_{\text{b}}\right)/\epsilon_{\text{d}}\right]$ , where *ω*_p_ denotes the bulk plasma frequency, *ϵ*_d_ is the permittivity of the surrounding dielectric medium, and *ϵ*_b_(*ω*) is the background permittivity accounting for the effective polarization from core electron screening in the metal – obtained here by subtracting the Drude free-electron contribution from experimental optical data according to the prescription of ref [51]. Remarkably, this simple treatment correctly describes the dispersion relation of SPPs in thin silver films comprised of *N* stacked (111) atomic planes, as depicted in Figure 1(a), even down to the few-atomic-layer regime. This is illustrated in Figure 1(b), where we superimpose the classical SPP dispersion curves *Q*_sp_ on a contour plot of the loss function Im{*r*_p_} of an ultrathin (*N* = 12) crystalline silver film, as predicted in a quantum-mechanical (QM) simulation of the associated reflection coefficient *r*_p_(*Q*, *ω*) (see below).

While the dominant features of the thin film optical response are captured in a classical (CL) approach, nonlocal and finite-size effects become increasingly important, particularly in the near field, as the film thickness decreases. In the linear regime, the near field response is naturally characterized by the Purcell factor [52] *P*(*ω*), which is defined as the ratio between the decay rate of an excited dipole emitter placed in the vicinity of the surface and its rate in free space. For an out-of-plane dipole, we have [30]

(1) $$P\left(\omega \right)=1+\frac{3c^{3}}{2\omega^{3}}\overset{\infty }{\underset{0}{\int }}\text{d}Q\;Q^{3}\;Im\left\{r_{\text{p}}\left(Q,\omega \right)\;{\text{e}}^{-2\kappa_{z}z_{0}}/\kappa_{z}\right\},$$

where $\kappa_{z}=\sqrt{Q^{2}-\omega^{2}/c^{2}+\text{i}0^{+}}$ (with the square root taken to yield Re{*κ*_*z*_} > 0). In what follows, we invoke the quasistatic limit by neglecting *ω*/*c* compared to *Q*, which is a reasonable approximation in view of the fact that the light wavelengths under consideration are large compared with the metal film thicknesses, and therefore, retardation corrections should only affect a negligible part of the integral in Eq. (1). Combining the above expression in the quasistatic limit with QM simulations of the reflection coefficient, we present in Figure 1(c) the Purcell factor for a dipole located at a distance *z*_0_ = 6 nm above the surface of Ag(111) films of thicknesses spanning *N* = 3 to *N* = 55 atomic planes. For thicker films, the linear scattering spectrum is found to exhibit a pronounced feature near the plasmon resonance that becomes sharper as the film thickness increases, eventually converging to a peak around *ℏω* ∼ 3.5 eV where the surface plasmon horizontal asymptotic feature dominates. However, for smaller thicknesses, the plasmon deviates further from the light line (overlapped with the vertical axis on the scale of Figure 1(b)) and thus makes more significant contributions over a wider frequency range.

Although the QM description of the linear optical response associated with plasmons in crystalline films does not deviate appreciably from classical predictions, the electronic band structure of ultrathin films is nonetheless rendered anharmonic by lateral quantum confinement. In a QM framework [53], quantized states emerge as solutions of the one-dimensional Schrödinger equation $H_{z}\varphi_{j}\left(z\right)=\hbar \varepsilon_{j}^{⊥}\varphi_{j}\left(z\right)$ for the Hamiltonian $H_{z}=-\hbar^{2}\partial_{z}^{2}/2m_{\text{e}}+V\left(z\right)$ , where *m*_e_ denotes the electron mass and *φ*_*j*_(*z*) are single-electron wave functions characterized by energies *ℏɛ*_*j*_ in the confinement direction, so that the total wave function $\Psi_{j,k_{∥}}=A^{-1/2}{\text{e}}^{\text{i}k_{∥}\cdot R}\varphi_{j}\left(z\right)$ exploits the translational invariance of a film (with normalization area A ) in the **R** = (*x*, *y*) plane. Importantly, electrons disperse in the QM model according to the relation $\hbar \varepsilon_{j,k_{∥}}=\hbar \varepsilon_{j}^{⊥}+\hbar^{2}k_{∥}^{2}/2m_{j}$ , with the second term accounting for free electron motion with 2D wave vector **k**_∥_ in the translationally invariant directions according to band-dependent effective masses *m*_*j*_, which have been shown to significantly impact the linear optical response of thin films [54]. Further details on the implementation can be found in the SI.

The anticipated increase in anharmonic valence electron motion with decreasing film thickness is illustrated in Figure 1(d) and (e), where we compare the density of states in momentum space for Ag(111) films in the semi infinite (*N* = 100, panel d) and few-atom-thickness (*N* = 5, panel e) regimes. More specifically, we compute the quantity

$$\underset{\hbar \varepsilon_{j}^{⊥}\leq E_{\text{F}}}{\sum }{\left|\int dz\;{\text{e}}^{-\text{i}k_{⊥}z}\varphi_{j}\left(z\right)\right|}^{2}L\left(\omega -\varepsilon_{j}^{⊥}\right)$$

from the wave functions *φ*_*j*_(*z*) decomposed in Fourier coefficients of the wave vector *k*_⊥_ along the film confinement direction, and weighted by a Lorentzian spectral distribution *L*(*ω*) of width *γ*, the inelastic scattering rate of conduction electrons (*ℏγ* = 21 meV for silver [55]). The spectrum of electronic states in the semi infinite film approximately follows the parabolic dispersion $\hbar^{2}k_{⊥}^{2}/2m_{\text{e}}$ (dashed curve), with a horizontal feature near the Fermi energy corresponding to the characteristic surface state of the (111) crystallographic surface. In contrast, the electronic spectrum of the *N* = 5 thin film is broken into discretized quantum-well states, along with features associated with the hybridization of (111) surface states across the film. Thus, electrons in ultrathin films are expected to undergo highly anharmonic motion in response to electromagnetic fields, favoring the emergence of strong *nonlinear* optical response.

### 2.1 Nonlinear response

**Figure 2:**
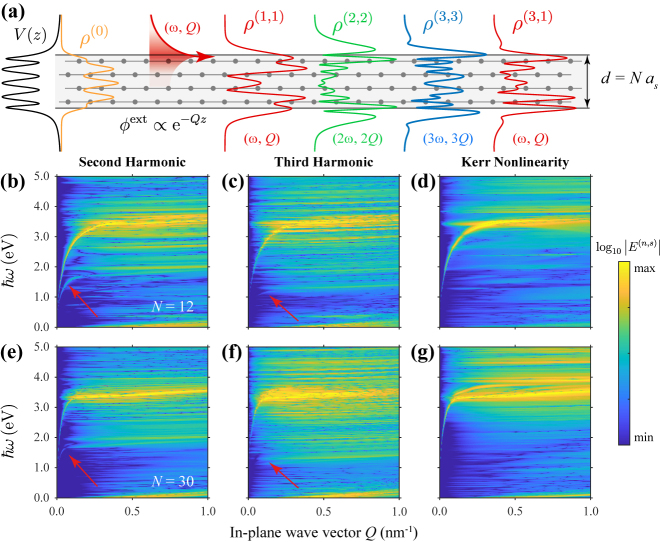
Nonlinear response of crystalline silver films. (a) Schematic illustration of the atomic layer potential *V*(*z*) (black curve), background electron density *ρ* ^(0)^ (orange curve), external potential with fixed frequency *ω* and in-plane wave vector *Q*, and induced charge density components *ρ* ^(*n*,*s*)^ corresponding to processes of order *n* and harmonic *s* in a film consisting of *N* = 4 Ag(111) atomic layers. We take *Q* = 0.1 nm^−1^ and *ℏω* = 1.85 eV to lie on the SPP curve. (b)–(g) Normal component of the induced fields $E_{z}^{\left(n,s\right)}$ associated with (b) and (e) SHG (*n* = *s* = 2), (b) and (f) third-harmonic generation (*n* = *s* = 3), and (d) and (g) the optical Kerr effect (*n* = 3, *s* = 1) as functions of in-plane wave vector and photon energy for films consisting of *N* Ag(111) atomic layers with (b)–(d) *N* = 12 and (e)–(g) *N* = 30.

We extend the linear QM model outlined above to describe nonlinear processes within the self-consistent field approximation, following a similar procedure as previously applied to nanostructured graphene [12, 56] (see details in Methods and SI). Aside from the linear contribution, the field induced at the surface by a nearby emitter has contributions **E** ^(*n*)^(*sω*) ≡ **E** ^(*n*,*s*)^ from nonlinear optical processes characterized by their perturbation order *n* = 1, 2, … and harmonic index *s* (with |*s*| ≤ *n*). Up to third order, these include linear response (*n* = *s* = 1), second- (*n* = *s* = 2) and third-harmonic (*n* = *s* = 3) generation, and an intensity-dependent correction to the response at the fundamental frequency from the optical Kerr effect (*n* = 3, *s* = 1). These processes are illustrated schematically in Figure 2(a) for evanescent fields at the surface of an ultrathin crystalline Ag(111) film with thickness *d* = *Na*_*s*_, where *N* denotes the number of stacked atomic planes and *a*_*s*_ = 0.236 nm the Ag(111) interlayer spacing. We consider *N* = 4 and also plot the atomic layer potential *V*(*z*) and the corresponding background charge distribution *ρ* ^(0)^, along with the external field and the induced charge densities *ρ* ^(*n*,*s*)^ for incident *Q* and *ω* within the SPP dispersion curve. Following the QM prescription in Methods to calculate the nonlinear induced fields, we find the results presented in Figure 2(b)–(g) as functions of optical in-plane wave vector *Q* and frequency *ω* for films consisting of *N* = 12 (Figure 2(b)–(d)) and *N* = 30 (Figure 2(e)–(g)) Ag(111) atomic layers. In all cases, prominent features in the generated nonlinear near field follow the plasmon dispersion curve *ω*_sp_(*Q*), with a more subtle peak emerging in second- and third-harmonic generation at *ω*_sp_(*Q*/2)/2 and *ω*_sp_(*Q*/3)/3, respectively (i.e., when the generated frequency matches the plasmon, indicated by red arrows). We note that vertical transitions between occupied and unoccupied bands result in weaker dispersionless features, which may be artificially enhanced by the assumption of parabolic bands in the QM model employed here [54]; signatures of such features with similar characteristics have been reported in experiment [57, 58], warranting further investigation with more rigorous treatments of the electronic band structure in future studies.

To quantify the strength of the plasmon-driven nonlinearity in the near field, we introduce a figure of merit (FoM) for evanescent fields,

(2) $$F^{\left(n,s\right)}\left(Q,\omega \right)=\frac{\phi^{\left(n,s\right)}\left(z_{0}\right)}{\phi_{0}\;{\text{e}}^{-\left(s+n\right)Q|z_{0}|}},$$

obtained from the ratio of the induced potential *ϕ* ^(*n*,*s*)^(*Q*, *z*_0_) = *∫* *d* *z* *v* ^(*s*)^(*Q*, *z*_0_, *z*)*ρ* ^(*n*,*s*)^(*Q*, *z*) (computed from the induced nonlinear charge density *ρ* ^(*n*,*s*)^ and evaluated at the source position *z* = *z*_0_) to the external potential $\propto {\text{e}}^{-nQ\left(z-z_{0}\right)}$ , and recognizing that the Coulomb interaction *v* ^(*s*)^(*Q*, *z*_0_, *z*) introduces an additional ${\text{e}}^{-sQz_{0}}$ factor at the frequency and wave vector generated by a nonlinear process of order *n* and harmonic *s*. In this manner, the surface nonlinearity for various thin films can be directly compared at specific frequencies and wave vector components, independently of the excitation origin *z*_0_.

**Figure 3:**
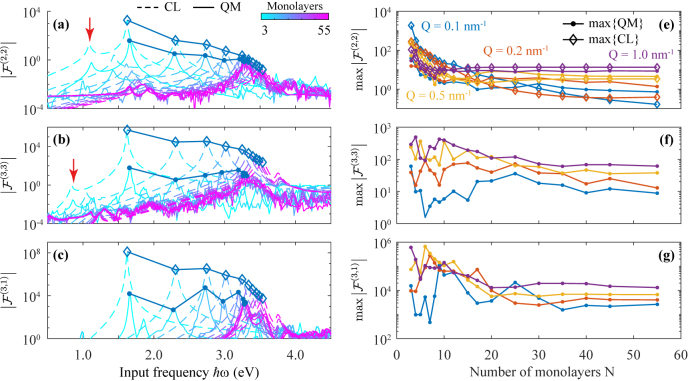
Nonlinear figure of merit. (a)–(c) Calculated figure of merit $F^{\left(n,s\right)}$ as a function of the input frequency with fixed in-plane wave vector *Q* = 0.1 nm^−1^ for (a) SHG, (b) THG, and (c) the Kerr effect. We consider a range of film thicknesses as indicated by the color scale. Solid and dashed curves correspond to QM and CL simulations, whose peak maxima are tracked with solid and open symbols, respectively. Red arrows in (a,b) highlight the SHG and THG replica of the plasmonic peak, equivalent to those of Figure 2. (e)–(g) Variation of the maxima of $F^{\left(n,s\right)}$ as a function of film thickness for a set of in-plane wave vectors (see colored labels in (e)).

In Figure 3 we apply the FoM to quantify second- and third-order nonlinear optical processes in Ag(111) films. Figure 3(a)–(c) shows SHG, THG, and the optical Kerr effect at a fixed in-plane wave vector *Q* = 0.1 nm^−1^ for various film thicknesses, as predicted from the QM model (solid curves) and contrasted with CL (dashed curves) predictions based on a purely two-dimensional treatment of ultrathin metal films (see Methods). As expected, we find that the response is dominated by the plasmonic peak, while its echoes in harmonic generation, indicated by the red arrows in Figures 2 and 3, appear with much lower intensity. The CL model predicts the spectral positions of the dominant features and reasonably captures their amplitudes for SHG. These maxima are featured in Figure 3(e)–(g) as functions of the number of atomic layers *N* at selected in-plane wave vectors *Q*, and are confirmed to generally increase with decreasing film thickness, particularly for films comprised of less than *N* = 20 atomic planes.

Inspecting Figure 3, we note that SHG undergoes a significant increase with decreasing film thickness, presumably due to the reliance of second-order optical nonlinearities on surface symmetry-breaking in an otherwise centrosymmetric medium, combined with the higher surface-to-volume ratio in thin films. In contrast, third-order processes dominated by the bulk response undergo a more modest increase in thinner films, and even dropping significantly in the Kerr nonlinearity for *N* ≲ 8. The differing nature of second- and third-order nonlinear processes in ultrathin films corroborates our finding that the CL method, based on a two-dimensional electron gas, cannot satisfactorily predict the amplitudes of THG and the Kerr nonlinearity. Additionally, the dimensionless quantity *Qd* characterizes the nonlocal response of the system and the spectral position of the plasmon, and consequently, the maxima in the nonlinear response for low in-plane momenta converge to their semi infinite values more slowly with thickness (e.g., comparing the blue and purple curves for *Q* = 0.1 nm^−1^ and *Q* = 1.0 nm^−1^, respectively). In general, excitation with small *Q* results in more dramatic changes in optical nonlinearity with respect to film thickness.

The CL model assuming a two-dimensional film is in good agreement with the QM model, but neglects finite-size effects, which become important for films comprised of *N* ≲ 20 atomic layers, as indicated by the additional features appearing in the spectra of Figure 3(a)–(c) in the QM model, originating in vertical single-electron transitions (see horizontal lines in Figure 2). The spectral positions of these features correlate with discrete levels in the electronic spectrum of ultrathin films, and thus vary dramatically with film thickness, leading to a complex dynamics for the prediction of the peaks, in agreement with experimental SHG measurements [38–41]. These findings underscore the importance of quantum finite-size effects in the thin film nonlinear response, which tend to cause increasing oscillatory behavior in the yield of higher-order processes, particularly those involving the generation of new frequencies, due to the involvement of additional vertical electronic transitions.

### 2.2 Manifestation of surface states in the nonlinear optical response

**Figure 4:**
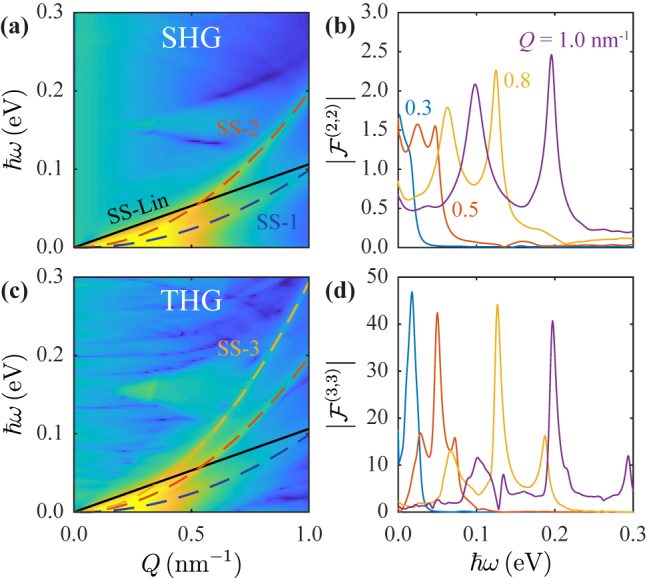
Shockley states in the nonlinear optical response. (a) Calculated dispersion diagram of SHG in the low *Q* and *ω* corner, revealing a nonlinear enhancement that involves Shockley states. The solid black line (SS-Lin) indicates the position of the SS acoustic plasmon demonstrated in the linear response and obtained from ref [59], whereas in SHG two resonances emanate at a different position than SS-Lin and are indicated by the dashed blue and red parabolic curves. (b) Low-frequency nonlinear FoM spectra for selected *Q*, indicated by the color-coded curves, in which surface-state features are clearly resolved. Panels (c) and (d) show the THG field and corresponding FoM in the same parameter space as panels (a) and (b), respectively. A third parabolic feature is indicated by the dashed yellow curve in (c). All curves are parametrized in Table 1.

The features emerging at low energies *ℏω* < 0.5 eV in Figures 1(b) and 2(b)–(g), which increase slowly with in-plane wave vector, originate in Shockley surface states (SSs) characteristic of the (111) metallic crystallographic orientation [60]. Unlike the weaker dispersionless features appearing in the nonlinear response due to vertical electronic transitions, the SSs are nearly independent of film thickness, each behaving as a two-dimensional electron gas that hybridizes with the bulk three-dimensional electron gas to produce intrinsic acoustic plasmons [59]. In Figure 4, we explore the low-energy peaks associated with SSs in the nonlinear optical response of thick films comprised of *N* = 55 Ag(111) atomic layers, which we regard as a well-converged limit representing a semi-infinite film. Specifically, we present the normal second-harmonic induced field in Figure 4(a), which reveals a broad, low-energy acoustic plasmon feature that rapidly disappears with increasing in-plane wave vector *Q*, presumably due to the onset of Landau damping. The acoustic plasmon is accompanied by two narrower features that persist at larger *Q*, which we attribute to single- and two-photon transitions involving the SSs. For comparison, we plot the linear acoustic dispersion (SS-Lin, black solid line) predicted in a previous work [59] and covering the energy range under consideration. In Figure 4(b), we compare the FoM for the SHG nonlinear response at selected values of *Q*, for which the SSs are clearly resolved at large in-plane wave vectors. Qualitatively similar behavior is observed for third-order nonlinear optical processes, as shown in Figure 4(c) and (d), where we present the nonlinear near field and FoM for THG, respectively. We note that a third parabolic feature emerges in the dispersion diagram of THG, corresponding to three-photon single-electron excitations involving the SSs. The commented SHG and THG response features are parametrized in Table 1 for THG and THG.

**Table 1:** Parametrization of linearly and parabolically dispersing resonances associated with Shockley surface states in the nonlinear optical response. We provide fitting parameters according to the expression *ℏω* = *aQ* ^2^ + *bQ*, also plotted in Figure 4. The SS-Lin line is taken from ref [59].

Label	*a* (eV nm^2^)	*b* (eV nm)
SS-Lin	–	0.1063
SS-1	0.103	−0.0042
SS-2	0.197	0.0006
SS-3	0.295	−0.0017

## 3 Concluding remarks

We have theoretically explored the nonlinear near-field optical response associated with plasmons in crystalline noble metal thin films. Our nonclassical approach, based on a phenomenological model that describes thin films composed of layered atomic planes, reveals a strong dependence on film thickness arising from quantum-confined states that emerges in the linear optical response and is amplified for nonlinear processes. The synergetic combination of thickness-dependent electronic structure and highly confined plasmonic excitations in ultrathin metal films endows them with excellent nonlinear optical properties that can be exploited for applications in near-field optics. The potentially longer-lived plasmons in high-quality crystalline films provide further motivation to explore these newly available quasi-2D materials as a platform for nanoscale nonlinear optics. Additionally, the enhanced sensitivity of nonlinear optical processes to finer details in the electronic structure of ultrathin films constitutes a powerful tool with which to probe and distinguish surface and bulk electronic properties of crystalline metals. In particular, we show that the Shockley states supported by the (111) crystalline metal surface can be more clearly resolved in the nonlinear optical response.

Detection of nonlinear far-field emission through near-field techniques like SNOM is challenging, but the radiation efficiency can be enhanced by dropping scatterers on the surface, either randomly or by arranging them in ordered arrays that would produce strong angular features. Lateral patterning of the metal thin film should also lead to preferential values of the in-plane wave vectors **Q** imposed by lateral confinement (e.g., the wave vector corresponding to a dipolar mode across a ribbon), for which the present theory should find direct application with those specific values of **Q**. Four-wave mixing constitutes another viable approach [61], while the combined use of electron and intense light pulses could provide a direct measurement of optical harmonic components [62]. We anticipate that our findings will stimulate future experimental efforts utilizing these and other approaches to study the nonlinear near-field response of plasmon polaritons in ultrathin crystalline metal films, circumventing the degradation of high-quality samples that is produced by patterning.

## 4 Methods

### 4.1 Near-field excitation by a point dipole

With near-field experiments in mind, we consider the external field produced by an electric dipole $p=-p\hat{z}$ oriented along the *z*-direction (e.g., representing an SNOM tip) a distance *z*_0_ > 0 above the upper surface of a thin film occupying the **R** = (*x*, *y*) plane and extending up to a distance *z* = *d*. Assuming a monochromatic time dependence e^−i*ωt*^ with frequency *ω* and working in the electrostatic limit, the external potential $\phi^{\text{ext}}\left(r,\omega \right)={\epsilon_{\text{d}}}^{-1}p\;\partial_{z}|r-z_{0}\hat{z}{|}^{-1}$ is decomposed in wave vector **Q** components using the Weyl identity according to

(3) $$\phi^{\text{ext}}\left(Q,\omega \right)=\frac{2\pi p}{\epsilon_{\text{d}}}{\text{e}}^{-Q|z-z_{0}|}\;\mathrm{sign}\left\{z_{0}-z\right\},$$

where *ϵ*_d_ is the dielectric function of the homogeneous medium that surrounds the film (we set *ϵ*_d_ = 1 for vacuum in the calculations here presented). The external potential excites the metal thin film, inducing fields that act back on the dipole (position **R** = 0, *z* = *z*_0_), where the self induced field is given by

(4) $$E_{z}^{\left(1,1\right)}\left(\omega \right)=-\int \frac{{\text{d}}^{2}Q}{{\left(2\pi \right)}^{2}}\partial_{z}\phi^{\left(1,1\right)}\left(Q,\omega \right).$$

Here, we have separated the induced potential *ϕ* ^(1,1)^(*Q*, *ω*) in **Q**-dependent components, which we compute following either classical (CL) or quantum-mechanical (QM) frameworks, as outlined below.

### 4.2 Electronic states of atomically-thin metal films

Following the prescription of ref [51], we consider a metal film of finite thickness *d* in the *z* direction, so that an electron propagating in the **R** = (*x*, *y*) plane with 2D momentum *ℏ* **k**_∥_ is characterized by the single-particle wave function $\Psi_{j,k_{∥}}\left(r\right)=A^{-1/2}{\text{e}}^{\text{i}k_{∥}\cdot R}\varphi_{j}\left(z\right)$ , with A denoting the quantization area and *φ*_*j*_(*z*) the out-of-plane wave function component obtained by solving the 1D Schrödinger equation $H_{0}\varphi_{j}\left(z\right)=\hbar \varepsilon_{j}^{⊥}\varphi_{j}\left(z\right)$ for the Hamiltonian $H_{0}=\left(-\hbar^{2}/2m_{\text{e}}\right)\partial_{z}^{2}+V\left(z\right)$ . The associated energy eigenvalues $\hbar \varepsilon_{j}^{⊥}$ of the 1D problem are supplemented in the full electron dispersion $\hbar \varepsilon_{j,k_{∥}}=\hbar \varepsilon_{j}^{⊥}+\hbar^{2}k_{\text{∥}}^{2}/2m_{j}$ by a parabolic term describing free-electron motion with effective mass *m*_*j*_ in directions parallel to the film surface. The choice of 1D potential *V*(*z*) determines the nature of the metal under consideration. We elaborate below and in the SI on the procedure used to determine the effective masses *m*_*j*_.

In the presence of the electrostatic potential *ϕ*, the electron dynamics is governed by the Liouville–von Neumann equation

(5) $$\text{i}\hbar \dot{\rho }=\left[H_{0}-e\phi ,\rho \right]-\text{i}\hbar \gamma \left(\rho -\rho^{\left(0\right)}\right),$$

supplemented by a term that accounts for relaxation of the density matrix *ρ* at a phenomenological rate *γ* to its *t* → −∞ equilibrium state $\rho^{\left(0\right)}\left(r,r^{\prime }\right)=\sum_{i}f_{i}\Psi_{i}\left(r\right)\Psi_{i}^{*}\left(r^{\prime }\right)$ , where *i* ≡ {*j*, **k**_∥_} denotes the multiplexed state index. Following Fermi–Dirac statistics at zero temperature, the equilibrium state is determined by the Fermi energy *E*_F_ through filling factors $f_{i}=\Theta \left(E_{\text{F}}-\hbar \varepsilon_{i}\right)$ , where Θ(*x*) denotes the step function. In practice, the Fermi energy is computed by populating states according to

$$\overset{M}{\underset{j=1}{\sum }}m_{j}^{*}\left(E_{\text{F}}-\hbar \varepsilon_{j}^{⊥}\right)=\pi \hbar^{2}n_{\text{eff}}d,$$

where *d* is the film thickness (e.g., *d* = *Na*_*s*_ for a film of *N* atomic layers with interlayer separation *a*_*s*_), *n*_eff_ is the effective electron density, and *M* is the highest partially occupied band, determined by the condition $\hbar \varepsilon_{M}^{⊥}<E_{\text{F}}<\hbar \varepsilon_{M+1}^{⊥}$ .

To compute the linear and nonlinear optical response of a metal film, we consider its interaction with an external potential *ϕ* ^ext^ that describes excitation by an evanescent field originating at a distance *z*_0_ above the film. Translational symmetry in **R** then facilitates the decomposition of *ϕ* ^ext^ in Fourier components of the in-plane optical wave vector **Q**. The optical response of conduction electrons in the film is characterized by the total electrostatic potential $\phi =\phi^{\text{ext}}+\phi_{\text{b}}^{\text{ind}}+v\cdot \rho^{\text{ind}}$ , where $\phi_{\text{b}}^{\text{ind}}$ accounts for the potential produced by a background induced polarization due to core electron screening, as discussed in ref [51], while the rightmost term in the potential results from the self-consistency of the induced charge,

$$\rho^{\text{ind}}\left(r,t\right)=-\frac{2e}{A}\underset{ii^{\prime }}{\sum }\left[\rho_{ii^{\prime }}\left(t\right)-\rho_{ii^{\prime }}^{\left(0\right)}\right]\Psi_{i}\left(r\right)\Psi_{i^{\prime }}\left(r\right),$$

mediated by the Coulomb interaction *v*(*Q*, *z*, *z*′). We use an analytical expression for *v* including the contribution of the polarization background [51], whereby the latter is calculated from the response of slab described by a local permittivity that is in turn obtained from the measured bulk dielectric function after eliminating the contribution of conduction electrons in the Drude model. The above expression is a sum over the diagonal elements of the real-space density matrix *ρ*(**r**, **r**, *t*) = ∑_*ii*_ *ρ*_*ii*_(*t*)Ψ_*i*_(**r**)Ψ_*i*_(**r**), expanded in the state basis {*i*, *i*′} with time-dependent matrix elements *ρ*_*ii*_(*t*), and including a factor of 2 to account for spin degeneracy.

### 4.3 Perturbative solution of the density matrix

Expanding the density matrix as a perturbation series in *ϕ* ^ext^, which is assumed to have a harmonic time dependence with frequency *ω*, we isolate the matrix elements of the *n*th-order contribution as

$$\rho_{jj^{\prime },k_{∥}k_{∥}^{\prime }}^{\left(n\right)}\left(t\right)=\overset{n}{\underset{s=-n}{\sum }}\rho_{jj^{\prime },k_{∥}}^{\left(n,s\right)}\delta_{k_{∥}^{\prime },k_{∥}-sQ}{\text{e}}^{-\text{i}s\omega t},$$

where, invoking in-plane momentum conservation, we have introduced the anzatz $\rho_{jj^{\prime },k_{∥}k_{∥}^{\prime }}^{\left(n,s\right)}\to \rho_{jj^{\prime },k_{∥}}^{\left(n,s\right)}\delta_{k_{∥}^{\prime },k_{∥}-sQ}$ , and have expanded in harmonics *s* up to |*s*| ≤ *n*. Given the self consistency of *ϕ* due to its dependence on *ρ*, we similarly write the potential as

$$\phi_{jj^{\prime },k_{∥}k_{∥}^{\prime }}^{\left(n\right)}=\overset{n}{\underset{s=-n}{\sum }}\phi_{jj^{\prime }}^{\left(n,s\right)}\delta_{k_{∥}^{\prime },k_{∥}-sQ}{\text{e}}^{-\text{i}s\omega t}.$$

Inserting the above expressions into Eq. (5) and equating terms of the same order *n* and harmonic *s*, we find a general expression for the matrix elements

(6) $$\rho_{jj^{\prime },k_{∥}}^{\left(n,s\right)}=-\frac{e}{\hbar }\frac{\left(f_{j^{\prime },k_{∥}-sQ}-f_{j,k_{∥}}\right)\phi_{jj^{\prime }}^{\left(n,s\right)}}{s\omega +\text{i}\gamma -\left(\varepsilon_{j,k_{∥}}-\varepsilon_{j^{\prime },k_{∥}-sQ}\right)}+\eta_{jj^{\prime },k_{∥}}^{\left(n,s\right)},$$

where the first term describes the contribution from the self-consistent potential and

$$\begin{array}{lc} \eta_{jj^{\prime },k_{∥}}^{\left(n,s\right)} & =-\frac{e}{\hbar }\sum_{n^{\prime }=1}^{n-1}\sum_{s^{\prime }=-n^{\prime }}^{n^{\prime }}\sum_{j^{\prime \prime }}\frac{\phi_{jj^{\prime \prime }}^{\left(n^{\prime },s^{\prime }\right)}\rho_{j^{\prime \prime }j^{\prime },k_{∥}-s^{\prime }Q}^{\left(n-n^{\prime },s-s^{\prime }\right)}-\phi_{j^{\prime \prime }j^{\prime }}^{\left(n^{\prime },s^{\prime }\right)}\rho_{jj^{\prime \prime },k_{∥}}^{\left(n-n^{\prime },s-s^{\prime }\right)}}{s\omega +\text{i}\gamma -\left(\varepsilon_{j,k_{∥}}-\varepsilon_{j^{\prime },k_{∥}-sQ}\right)} \end{array}$$

acts as a source term constructed from the response at lower perturbation orders. The induced charge density characterizing the optical response at order *n* and harmonic *s* is constructed according to

$$\rho^{\text{ind}}\left(r,t\right)=-2e\underset{n,s}{\sum }\frac{1}{A}\underset{jj^{\prime },k_{∥}}{\sum }\rho_{jj^{\prime },k_{∥}}^{\left(n,s\right)}\varphi_{j}\left(z\right)\varphi_{j^{\prime }}\left(z\right){\text{e}}^{\text{i}s\left(Q\cdot R-\omega t\right)}.$$

In practice, we compute the matrix elements $\rho_{jj^{\prime }k_{∥}}^{\left(n,s\right)}$ by projecting in sinusoidal basis functions *s*_*l*_(*z*) ≡ (2/*L*)^−1/2^ sin(*πlz*/*L*), indexed by *l* = 1, 2, … and spanning a simulation domain in *z* of width *L* > *d* = *Na*_*s*_ extending beyond the actual film thickness. The induced charge density can be expressed in this basis as $\sum_{l}\rho_{l}^{\left(n,s\right)}s_{l}\left(z\right){\text{e}}^{\text{i}s\left(Q\cdot R-\omega t\right)}$ , with coefficients

$$\rho_{l}^{\left(n,s\right)}=-2e\underset{jj^{\prime }}{\sum }s_{l,jj^{\prime }}\int \frac{\text{d}k_{∥}}{{\left(2\pi \right)}^{2}}\rho_{jj^{\prime },k_{∥}}^{\left(n,s\right)},$$

where we have defined $s_{l,jj^{\prime }}\equiv \int dz\;s_{l}\left(z\right)\varphi_{j}\left(z\right)\varphi_{j^{\prime }}^{*}\left(z\right)$ . We obtain $\rho_{l}^{\left(n,s\right)}$ by expressing Eq. (6) in terms of matrices indexed by *l* and *l*′ as

$$\rho^{\left(n,s\right)}=\chi^{\left(0,s\right)}\cdot {\left[1-v^{\left(s\right)}\chi^{\left(0,s\right)}\right]}^{-1}\cdot \beta^{\left(n,s\right)}+\eta^{\left(n,s\right)},$$

where

$$\begin{array}{ll} v_{ll^{\prime }}^{\left(s\right)} & =\int dz\int dz^{\prime }s_{l}\left(z\right)s_{l^{\prime }}\left(z^{\prime }\right)v\left(sQ,z,z^{\prime }\right),\\ \eta_{l}^{\left(n,s\right)} & =-2e\underset{jj^{\prime }}{\sum }s_{l,jj^{\prime }}\int \frac{\text{d}k_{∥}}{{\left(2\pi \right)}^{2}}\eta_{jj^{\prime },k_{∥}}^{\left(n,s\right)}, \end{array}$$

and

$$\begin{array}{ll} \chi_{ll^{\prime }}^{\left(0,s\right)} & =\frac{2{\text{e}}^{2}}{\hbar }\underset{jj^{\prime }}{\sum }\int \frac{\text{d}k_{∥}}{{\left(2\pi \right)}^{2}}\left(f_{j^{\prime },k_{∥}-sQ}-f_{j,k_{∥}}\right)\\ & \;\times \frac{s_{l,jj^{\prime }}s_{l^{\prime },jj^{\prime }}^{*}}{s\omega +\text{i}\gamma -\left(\varepsilon_{j,k_{∥}}-\varepsilon_{j^{\prime },k_{∥}-sQ}\right)} \end{array}$$

is the noninteracting RPA susceptibility evaluated at frequency *sω* and optical wave vector *s* **Q**, while

$$\beta_{l}^{\left(n,s\right)}=\left(\phi_{l}^{\text{ext}}+\phi_{b,l}^{\text{ind}}\right)\delta_{n,1}\left(\delta_{s,-1}+\delta_{s,1}\right)+\underset{l^{\prime }}{\sum }v_{ll^{\prime }}^{\left(s\right)}\eta_{l^{\prime }}^{\left(n,s\right)}$$

is the source potential expressed in the sinusoidal basis, with the external part contributing only to drive the linear response. Further details on the perturbative approach outlined above are provided in the SI.

### 4.4 Determination of the electron effective mass

In the QM model, we characterize in-plane electron motion in the quantized state *j* by the parabolic dispersion $\hbar \varepsilon_{j,k_{∥}}=\hbar \varepsilon_{j}^{⊥}+\hbar^{2}k_{\text{∥}}^{2}/2m_{j}$ , adopting band-dependent electron effective masses *m*_*j*_. These effective masses are fitted to *ab-initio* band structure calculations using the Vienna *ab initio* simulation package (VASP) [63–65]. More specifically, the effective mass *m*_*j*_ of state *j* is obtained by fitting $\hbar k_{\text{∥}}^{2}/2m_{j}$ to the dispersion of that state at the Fermi level *E*_F_ (i.e., in the neighborhood of electronic transitions dominating the low-energy plasmonic response), since plasmons are primarily comprised of virtual electron–hole pair transitions among one-electron states with small separation compared with the plasmon energy [36]. It should be noted that, while the first principles electronic bands are well captured by fitting parabolas within a few eV of the Fermi level, the bands deviate significantly from parabolic profiles at energies beyond this range, where they affect negligibly the optical response for the range of optical energy and momentum under consideration. Further details on the implementation of the numerical simulation can be found in the SI.

### 4.5 Classical model of a two-dimensional film

In a classical quasistatic framework, we approximate the ultrathin film as a two-dimensional layer, for which the *n*th-order optical response generating a harmonic *s* with in-plane wave vector component **Q** and frequency *ω* is characterized by an induced charge density *ρ* ^(*n*,*s*)^(*z*) = *ρ* ^(*n*,*s*)^ *δ*(*z*) (i.e., in the *z* = 0 plane) that generates the potential *ϕ* ^(*n*,*s*)^(*z*) = *∫*d*z*′*v* ^(*s*)^(*z*, *z*′)*ρ* ^(*n*,*s*)^(*z*′) = (2*π*/*sQ*)e^−*sQ*|*z*|^ *ρ* ^(*n*,*s*)^. Invoking the continuity equation ∇ ⋅**J** ^(*n*,*s*)^ + ∂_*t*_ *ρ* ^(*n*,*s*)^ = 0, we can then write the potential as

(7) $$\phi^{\left(n,s\right)}\left(z\right)=\frac{2\pi }{sQ}{\text{e}}^{-sQ|z|}{\left[\frac{1}{s\omega }\nabla \cdot J^{\left(n,s\right)}\right]}_{z=0},$$

where **J** ^(*n*,*s*)^ is the total induced surface current.

To linear order, the total potential resulting from an evanescent source with amplitude *ϕ*_0_ located at *z*_0_ > 0 is

$$\phi^{\left(1,1\right)}\left(z\right)=\phi_{0}\left[{\text{e}}^{-Q\left(z-z_{0}\right)}-r_{\text{p}}\left(Q,\omega \right){\text{e}}^{-Q\left(|z|+z_{0}\right)}\right]$$

where *r*_p_(*Q*, *ω*) is the reflection coefficient of the film, obtained in the Fabry–Pérot formalism as

$$r_{\text{p}}\left(\omega ,Q\right)=r_{\text{dm}}+\frac{t_{\text{dm}}t_{\text{md}}r_{\text{md}}{\text{e}}^{-2Qd}}{1-r_{\text{md}}r_{\text{md}}{\text{e}}^{-2Qd}},$$

with $r_{\text{dm}}=-r_{\text{md}}=\left(\epsilon_{\text{m}}-\epsilon_{d}\right){\left(\epsilon_{\text{m}}+\epsilon_{d}\right)}^{-1}$ and $t_{\text{dm}}t_{\text{md}}=4\epsilon_{d}\epsilon_{\text{m}}{\left(\epsilon_{\text{m}}+\epsilon_{d}\right)}^{-2}$ . In the calculations here presented, we consider self standing films (i.e., *ϵ*_*d*_ = 1) and take the dielectric function of the metal from experimental values [55].

For nonlinear optical processes, we construct the current **J** = ∂_*t*_ **P** from the polarization $P^{\left(n,s\right)}=\chi^{\left(1,s\right)}E^{\left(n,s\right)}+P_{\text{NL}}^{\left(n,s\right)}$ , where the first term accounts for the linear response of the film (via the linear susceptibility *χ* ^(1,*s*)^) to the nonlinear induced field and the second term is the source of the nonlinearity, proportional to the nonlinear susceptibility *χ* ^(*n*,*s*)^, and more explicitly, given by

$$\begin{array}{ll} P_{z}^{\left(2,2\right)} & =\chi_{\text{zzz}}^{\left(2\right)}\left(\omega \right){\left[E_{z}^{\left(1,1\right)}\right]}^{2}\\ P^{\left(3,3\right)} & ={\tilde{\chi }}^{\left(3\right)}\left(\omega \right){\left[E^{\left(1,1\right)}\right]}^{3}\\ P^{\left(3,1\right)} & ={\tilde{\chi }}^{\left(3\right)}\left(\omega \right)\left\{E^{\left(1,-1\right)}{\left[E^{\left(1,1\right)}\right]}^{2}+2E^{\left(1,1\right)}|E^{\left(1,1\right)}{|}^{2}\right\} \end{array}$$

for SHG, THG, and Kerr nonlinearity. The associated potential is then given from Eq. (7) as

$$\phi^{\left(n,s\right)}\left(z\right)=\frac{2\pi }{sQ}{\text{e}}^{-sQ|z|}\left[1-r_{\text{p}}\left(sQ,s\omega \right)\right]\eta_{\text{CL}}^{\left(n,s\right)},$$

where $\eta_{\text{CL}}^{\left(n,s\right)}$ plays a similar role to that in Eq. (6) in the QM model, and is obtained for SHG, THG, and the Kerr nonlinearity as

$$\begin{array}{ll} \eta_{\text{CL}}^{\left(2,2\right)} & =2Q^{3}\chi_{\text{zzz}}^{\left(2,2\right)}{\left[\phi_{z=0}^{\left(1,1\right)}\right]}^{2},\\ \eta_{\text{CL}}^{\left(3,3\right)} & =3Q^{4}{\tilde{\chi }}^{\left(3,3\right)}{\left[\phi_{z=0}^{\left(1,1\right)}\right]}^{3},\\ \eta_{\text{CL}}^{\left(3,1\right)} & =-3Q^{4}{\tilde{\chi }}^{\left(3,1\right)}{\left|\phi_{z=0}^{\left(1,1\right)}\right|}^{2}\phi_{z=0}^{\left(1,1\right)}, \end{array}$$

with ${\tilde{\chi }}^{\left(n,s\right)}=\chi^{\left(n,s\right)}d$ incorporating the film thickness *d*. For SHG, the use of a two-dimensional model is meaningful because only the surface contributes due to the centrosymmetry of the bulk metal; here, we use a local surface second-order susceptibility of the form

(8) $$\chi_{\text{zzz}}^{\left(2\right)}\left(\omega \right)=a\left(\omega \right)\frac{e}{16\pi m_{\text{e}}}\frac{\epsilon_{\text{m}}\left(\omega \right)-1}{\omega^{2}},$$

where *e* > 0 and *m*_e_ are the electron charge and mass, respectively, and *a*(*ω*) is a standard nonlinear parameter, for which we adopt the dispersionless mean value *a* = 7.75 reported in ref [66].

To describe third-order processes, we also use a 2D susceptibility and incorporate the reported bulk value [2] *χ* ^(3)^ = 2.9 × 10^−19^ V^2^/m^2^. Note that this model for third order processes is dispersionless, neglects nonlocality, and further cannot account for (weak) cascaded nonlinear processes such as ∝ *ϕ* ^22^ *ϕ* ^11^, which are included to the QM model. However, it should be reasonable when the response is dominated by plasmons at the fundamental frequency.

## Supplementary Material

Supplementary Material Details
