# Supplementary material for: Nonlinear plasmonic response in atomically thin metal films
Source: Nanophotonics. 2021 Oct 15;10(16):4149–59. doi: 10.1515/nanoph-2021-0422 (PMC9651024; doi:10.1515/nanoph-2021-0422)
Supplement: Supplementary file 1 — Supplementary Material Details [file j_nanoph-2021-0422_suppl.pdf]

Nonlinear plasmonic response in atomically thin metal films

– SUPPLEMENTARY INFORMATION –

A. Rodríguez Echarri,[1] Joel D. Cox,[2,3,*] F. Iyikanat,[1] and F. Javier García de Abajo[1,4,†]

[1] *ICFO-Institut de Ciencies Fotoniques, The Barcelona Institute of Science and Technology, 08860 Castelldefels (Barcelona), Spain*

[2] *Center for Nano Optics, University of Southern Denmark, Campusvej 55, DK-5230 Odense M, Denmark*

[3] *Danish Institute for Advanced Study, University of Southern Denmark, Campusvej 55, DK-5230 Odense M, Denmark*

[4] *ICREA-Institució Catalana de Recerca i Estudis Avançats, Passeig Lluís Companys 23, 08010 Barcelona, Spain*

(Dated: September 22, 2021)

We provide a detailed derivation of the main equations used in the main text, based on a classical electrostatic description of the fields, a self-consistent treatment of conduction electrons in the films, and perturbation theory beyond linear order to compute each of the nonlinear optical response functions. Additional details on the calculation of the effective masses are also provided.

Contents

S1. Electronic states and density matrix 1

S2. Perturbation series expansion 2
- A. Linear response 2
- B. Nonlinear response 2
- C. Self-consistent optical response calculations through expansion in an orthonormal basis 3

S3. Electronic structure of crystalline metal films 4
- A. Atomic layer potential 4
- B. Characterization of effective masses 5
- C. First-principles description of effective masses 5

References 6

S1. ELECTRONIC STATES AND DENSITY MATRIX

We consider a metallic film that is translationally invariant in the $\mathbf{R}=(x,y)$ plane and finite in the z-direction, so that electrons in the unperturbed film are confined by a potential $V(z)$ entering the Hamiltonian

$$\mathcal{H}_0=-\frac{\hbar^2}{2m_{\rm e}}\nabla^2+V(z) \tag{S1}$$

which admits a complete set of separable wave functions

$$\Psi_{j,\mathbf{k}_\parallel}(\mathbf{r})=\frac{e^{{\rm i}\mathbf{k}_\parallel\cdot\mathbf{R}}}{\sqrt{\mathcal{A}}}\varphi_j(z), \tag{S2}$$

where $\mathcal{A}$ is a normalization area, $\hbar\mathbf{k}_\parallel$ is the in-plane electron momentum, and $\varphi_j(z)$ are localized wave functions satisfying

$$\left[-\frac{\hbar^2}{2m_{\rm e}}\frac{\partial^2}{\partial z^2}+V(z)\right]\varphi_j(z)=\hbar\varepsilon_j^\perp\varphi_j(z), \tag{S3}$$

from which the obtained energy eigenvalues $\hbar\varepsilon_j^\perp$ contribute to the total electron energy $\hbar\varepsilon_{j,\mathbf{k}_\parallel}=\hbar\varepsilon_j^\perp+\hbar^2k_\parallel^2/2m_j$ after having introduced band-dependent effective masses m_j to the parabolic in-plane dispersion.

In the quasistatic approximation, the thin film interacts with an electrostatic potential $\phi=\phi^{\rm ext}+\phi^{\rm ind}_{\rm b}+\phi^{\rm ind}$, comprised of the external perturbation

$$\phi^{\rm ext}(\mathbf{r},t)=\phi^{\rm ext}(z){\rm e}^{{\rm i}(\mathbf{Q}\cdot\mathbf{R}-\omega t)}+{\rm c.c.}, \tag{S4}$$

a background polarization potential due to core electron screening, as reported in Ref. 1, and the induced potential

$$\phi^{\rm ind}(\mathbf{r},t)=\int d^3\mathbf{r}'\,\frac{\rho^{\rm ind}(\mathbf{r}',t)}{|\mathbf{r}-\mathbf{r}'|} \tag{S5}$$

arising from the polarization of conduction electrons. We characterize the external potential by a fixed in-plane wave vector component $\mathbf{Q}$ and frequency ω, while the induced potential is written in terms of the induced charge $\rho^{\rm ind}(\mathbf{r},t)=-2e[\rho(\mathbf{r},\mathbf{r},t)-\rho^{(0)}(\mathbf{r},\mathbf{r})]$, which is in turn obtained from the diagonal elements of the single-particle density matrix

$$\rho(\mathbf{r},\mathbf{r}',t)=\sum_{jj',\mathbf{k}_\parallel\mathbf{k}'_\parallel}\rho_{jj',\mathbf{k}_\parallel\mathbf{k}'_\parallel}(t)\Psi_{j,\mathbf{k}_\parallel}(\mathbf{r})\Psi^*_{j',\mathbf{k}'_\parallel}(\mathbf{r}'), \tag{S6}$$

where $\rho_{jj',\mathbf{k}_\parallel\mathbf{k}'_\parallel}(t)$ are time-dependent coefficients, the dynamics of which is governed by the Liouville von Neumann equation

$${\rm i}\hbar\frac{\partial\rho}{\partial t}=[\mathcal{H}_0-e\phi,\rho]-{\rm i}\hbar\gamma\left(\rho-\rho^{(0)}\right). \tag{S7}$$

The above expression incorporates inelastic electron scattering by relaxing the density matrix at a phenomenological rate γ to its equilibrium value

$$\rho^{(0)}(\mathbf{r},\mathbf{r}')=\sum_{j,\mathbf{k}_\parallel}f_{j,\mathbf{k}_\parallel}\Psi_{j,\mathbf{k}_\parallel}(\mathbf{r})\Psi^*_{j,\mathbf{k}_\parallel}(\mathbf{r}'), \tag{S8}$$

*Electronic address: cox@mci.sdu.dk
†Electronic address: javier.garciadeabajo@nanophotonics.es

where $f_{j,\mathbf{k}_\parallel}$ are occupation factors following Fermi-Dirac statistics. In practice we adopt the zero-temperature limit, so that $f_{j,\mathbf{k}_\parallel} = \Theta(E_\mathrm{F} - \varepsilon_{j,\mathbf{k}_\parallel})$, with $\Theta(x)$ denoting the Heaviside step function and E_F the Fermi energy.

S2. PERTURBATION SERIES EXPANSION

Inserting Eq. (S6) into Eq. (S7), we obtain

$$\begin{aligned}&\left[\mathrm{i}(\partial_t + \gamma) - (\varepsilon_i - \varepsilon_{i'})\right]\rho_{ii'} = \\ &-\frac{e}{\hbar}\sum_{i''}\left(\phi_{ii''}\rho_{i''i'} - \rho_{ii''}\phi_{i''i'}\right) + \mathrm{i}\gamma f_i\delta_{ii'},\end{aligned} \tag{S9}$$

where $i = \{j, \mathbf{k}_\parallel\}$ denotes a multiplexed state index that simplifies the notation, and the matrix elements of the potential are

$$\phi_{ii'} = \frac{1}{\mathcal{A}}\int d^3\mathbf{r}\,\phi(\mathbf{r},t)\mathrm{e}^{-\mathrm{i}(\mathbf{k}_\parallel - \mathbf{k}'_\parallel)\cdot\mathbf{R}}\varphi_j(z)\varphi_{j'}(z). \tag{S10}$$

The density matrix and total potential are each expanded in perturbation series with respect to the external potential according to

$$\rho = \rho^{(0)} + \rho^{(1)} + \ldots = \sum_{n=0}^{\infty}\rho^{(n)}, \quad \phi = \sum_{n=1}^{\infty}\phi^{(n)}, \tag{S11}$$

where the latter is obtained from the former through Eq. (S5). Inserting Eq. (S11) into Eq. (S9) and equating terms of equal perturbation order, we find

$$\begin{aligned}&\left[\mathrm{i}(\partial_t + \gamma) - (\varepsilon_i - \varepsilon_{i'})\right]\rho^{(n)}_{ii'} - \mathrm{i}\gamma f_i\delta_{ii'} = \\ &-\frac{e}{\hbar}\sum_{n'=1}^{\infty}\sum_{i''}\left[\phi^{(n')}_{ii''}\rho^{(n-n')}_{i''i'} - \rho^{(n-n')}_{ii''}\phi^{(n')}_{i''i'}\right],\end{aligned} \tag{S12}$$

which reveals that the equation of motion for the density matrix at perturbation order n is constructed according to

$$\rho^{(n)} \propto \sum_{n'=1}^{n}\phi^{(n')}\rho^{(n-n')}$$

(i.e., self-consistently through the induced potential). The latter depends on the density matrix through the induced charge, and also trough the density matrix obtained at lower perturbation orders $n' < n$. We note that the right-hand side of Eq. (S12) disappears at $n = 0$ order, thus reducing the expression to $\rho^{(0)}_{ii'} = f_i\delta_{ii'}$ and satisfying Eq. (S8).

A. Linear response

To first order, the right-hand side of Eq. (S12) only has a contribution $\propto \phi^{(1)}\rho^{(0)}$, while the external potential in Eq. (S4) depends on in-plane position and time according to $\mathrm{e}^{\mathrm{i}s(\mathbf{Q}\cdot\mathbf{R}-\omega t)}$, where $s = \pm 1$ is the harmonic index. Then, the integral over $\mathbf{R}$ in Eq. (S10) for ϕ^{ext} effectively yields a term for each s proportional to the Kronecker delta $\delta_{\mathbf{k}'_\parallel,\mathbf{k}_\parallel - s\mathbf{Q}}$, directly enforcing parallel momentum conservation, and containing a time dependence $\mathrm{e}^{-\mathrm{i}s\omega t}$. We thus conclude that the density matrix at linear order in the external potential inherits the same momentum and frequency dependence, allowing us to write

$$\rho^{(1)}_{jj',\mathbf{k}_\parallel\mathbf{k}'_\parallel} = \sum_{s=\pm1}\rho^{(1,s)}_{jj',\mathbf{k}_\parallel}\delta_{\mathbf{k}'_\parallel,\mathbf{k}_\parallel - s\mathbf{Q}}\mathrm{e}^{-\mathrm{i}s\omega t} \tag{S13}$$

and

$$\phi^{(1)}_{jj',\mathbf{k}_\parallel\mathbf{k}'_\parallel} = \sum_{s=\pm1}\phi^{(1,s)}_{jj'}\delta_{\mathbf{k}'_\parallel,\mathbf{k}_\parallel - s\mathbf{Q}}\mathrm{e}^{-\mathrm{i}s\omega t}. \tag{S14}$$

Inserting the above expression for the total potential into Eq. (S10), we isolate the coefficients

$$\phi^{(1,s)}_{jj'} = \int dz\,\varphi^*_j(z)\varphi_{j'}(z)\phi^{(1,s)}(z),$$

where

$$\begin{aligned}\phi^{(1,s)}(z) =&\phi^{\mathrm{ext}}(z) + \phi^{\mathrm{ind}}_{\mathrm{b}}(z)\\ &+ \int dz' v^{(s)}(z,z')\rho^{\mathrm{ind},(1,s)}(z').\end{aligned} \tag{S15}$$

Here, we have introduced the $s\mathbf{Q}$ in-plane Fourier component of the Coulomb interaction $v^{(s)}(z,z')$ and the linear induced charge density at harmonic s

$$\rho^{\mathrm{ind},(1,s)}(z) = -\frac{2e}{\mathcal{A}}\sum_{jj',\mathbf{k}_\parallel}\rho^{(1,s)}_{jj',\mathbf{k}_\parallel}\varphi_j(z)\varphi^*_{j'}(z).$$

We now insert Eq. (S13) into Eq. (S12) to isolate the coefficients

$$\rho^{(1,s)}_{jj',\mathbf{k}_\parallel} = -\frac{e}{\hbar}\frac{f_{j',\mathbf{k}_\parallel - s\mathbf{Q}} - f_{j,\mathbf{k}_\parallel}}{s\omega + \mathrm{i}\gamma - \left(\varepsilon_{j,\mathbf{k}_\parallel} - \varepsilon_{j',\mathbf{k}_\parallel - s\mathbf{Q}}\right)}\phi^{(1,s)}_{jj'},$$

from which the linear induced charge density is obtained.

B. Nonlinear response

The method employed in the previous section to extract the linear response of the thin film apply generally to nonlinear processes of order $n > 1$ and harmonic index s, with $|s| \leq n$. In particular, following Eq. (S13), we write

$$\rho^{(n)}_{jj',\mathbf{k}_\parallel\mathbf{k}'_\parallel} = \sum_{s=-n}^{n}\rho^{(n,s)}_{jj',\mathbf{k}_\parallel}\delta_{\mathbf{k}'_\parallel,\mathbf{k}_\parallel - s\mathbf{Q}}\mathrm{e}^{-\mathrm{i}s\omega t} \tag{S16}$$

and

$$\phi^{(n)}_{jj',\mathbf{k}_\parallel\mathbf{k}'_\parallel} = \sum_{s=-n}^{n}\phi^{(n,s)}_{jj'}\delta_{\mathbf{k}'_\parallel,\mathbf{k}_\parallel - s\mathbf{Q}}\mathrm{e}^{-\mathrm{i}s\omega t}, \tag{S17}$$

which upon insertion into Eq. (S10) yields the matrix elements

$$\phi^{(n,s)}_{jj'} = \int dz\, \varphi^*_j(z)\varphi_{j'}(z)\phi^{(n,s)}(z). \tag{S18}$$

Here,

$$\phi^{(n,s)}(z) = \left[\phi^{\rm ext}(z) + \phi^{\rm ind}_{\rm b}(z)\right]\delta_{n,1}\left(\delta_{s,-1}+\delta_{s,1}\right) + \int dz\, v^{(s)}(z,z')\rho^{{\rm ind},(n,s)}(z') \tag{S19}$$

and

$$\rho^{{\rm ind},(n,s)}(z) = -2e \sum_{jj',\mathbf{k}_\parallel} \rho^{(n,s)}_{jj',\mathbf{k}_\parallel}\varphi_j(z)\varphi^*_{j'}(z) \tag{S20}$$

are the amplitudes of the potential and induced charge, respectively, which depend on the film confinement coordinate z.

Following analogous steps as those used to obtain the first-order density matrix, Eq. (S12) becomes

$$\rho^{(n,s)}_{jj'\mathbf{k}_\parallel} = -\frac{e}{\hbar}\sum_{n'=1}^{n}\sum_{s'=-n'}^{n'} \times \sum_{j''} \frac{\phi^{(n',s')}_{jj''}\rho^{(n-n',s-s')}_{j''j',\mathbf{k}_\parallel-s'\mathbf{Q}} - \rho^{(n-n',s-s')}_{jj'',\mathbf{k}_\parallel}\phi^{(n',s')}_{j''j'}}{s\omega + {\rm i}\gamma - \left(\varepsilon_{j,\mathbf{k}_\parallel} - \varepsilon_{j',\mathbf{k}_\parallel-s\mathbf{Q}}\right)}, \tag{S21}$$

where the matrix elements of the potential are

$$\phi^{(n,s)}_{jj'} = \int dz\, \varphi^*_j(z)\varphi_{j'}(z)\phi^{(n,s)}(z). \tag{S22}$$

We now follow a computationally efficient method to calculate $\rho^{(n,s)}$ by first isolating the self-consistent term in the summation of Eq. (S21) according to

$$\rho^{(n,s)}_{jj',\mathbf{k}_\parallel} = -\frac{e}{\hbar}\frac{\left(f_{j',\mathbf{k}_\parallel-s\mathbf{Q}} - f_{j,\mathbf{k}_\parallel}\right)\phi^{(n,s)}_{jj'}}{s\omega + {\rm i}\gamma - \left(\varepsilon_{j,\mathbf{k}_\parallel} - \varepsilon_{j',\mathbf{k}_\parallel-s\mathbf{Q}}\right)} + \eta^{(n,s)}_{jj',\mathbf{k}_\parallel}, \tag{S23}$$

where we have used Eq. (S8) to write $\rho^{(0)}_{jj',\mathbf{k}_\parallel} = \delta_{jj'}f_{j,\mathbf{k}_\parallel}$ and defined

$$\eta^{(n,s)}_{jj',\mathbf{k}_\parallel} = -\frac{e}{\hbar}\sum_{n'=1}^{n-1}\sum_{s'=-n'}^{n'} \times \sum_{j''} \frac{\phi^{(n',s')}_{jj''}\rho^{(n-n',s-s')}_{j''j',\mathbf{k}_\parallel-s'\mathbf{Q}} - \phi^{(n',s')}_{j''j'}\rho^{(n-n',s-s')}_{jj'',\mathbf{k}_\parallel}}{s\omega + {\rm i}\gamma - \left(\varepsilon_{j,\mathbf{k}_\parallel} - \varepsilon_{j',\mathbf{k}_\parallel-s\mathbf{Q}}\right)}. \tag{S24}$$

The latter collects the dependence on perturbation orders $n' < n$, which drive the nonlinear response.

C. Self-consistent optical response calculations through expansion in an orthonormal basis

In practical calculations, we expand quantities depending on z in a complete basis set of orthonormalized sine functions

$$s_l(z) \equiv \sqrt{\frac{2}{L}}\sin\left(\frac{l\pi z}{L}\right), \tag{S25}$$

where l is a positive integer and L is the length of the simulation domain. In terms of the above basis functions, we write the induced charge as

$$\rho^{{\rm ind},(n,s)}(z) = \sum_l \rho^{(n,s)}_l s_l(z), \tag{S26}$$

where the expansion coefficients are

$$\rho^{(n,s)}_l = -2e\sum_{jj'}\int dz\, s_l(z)\varphi_j(z)\varphi^*_{j'}(z)\int\frac{d\mathbf{k}_\parallel}{(2\pi)^2}\rho^{(n,s)}_{jj',\mathbf{k}_\parallel}. \tag{S27}$$

Here, we have transformed the sum over $\mathbf{k}_\parallel$ into an integral according to the prescription $\mathcal{A}^{-1}\sum_{\mathbf{k}_\parallel} \to \int d\mathbf{k}_\parallel/(2\pi)^2$. In a similar fashion, we write the potential as

$$\phi^{(n,s)}(z) = \sum_l \phi^{(n,s)}_l s_l(z), \tag{S28}$$

where

$$\phi^{(n,s)}_l = \sum_{jj'}\int dz\, s_l(z)\varphi_j(z)\varphi^*_{j'}(z)\phi^{(n,s)}_{jj'}. \tag{S29}$$

Then, expressing the matrix elements of the potential in Eq. (S22) as

$$\phi^{(n,s)}_{jj'} = \sum_l \phi^{(n,s)}_l \int dz\, s_l(z)\varphi^*_j(z)\varphi_{j'}(z) = \sum_l s_{l,jj'}\phi^{(n,s)}_l \tag{S30}$$

with $s_{l,jj'} \equiv \int dz\, s_l(z)\varphi^*_j(z)\varphi_{j'}(z)$, we can write Eq. (S23) in terms of the $s_l(z)$ functions as

$$\rho^{(n,s)}_l = \sum_{l'}\chi^{(0,s)}_{ll'}\phi^{(n,s)}_{l'} + \eta^{(n,s)}_l, \tag{S31}$$

where

$$\eta^{(n,s)}_l = -2e\sum_{jj'}s_{l,jj'}\int\frac{d\mathbf{k}_\parallel}{(2\pi)^2}\eta^{(n,s)}_{jj',\mathbf{k}_\parallel} \tag{S32}$$

and

$$\chi^{(0,s)}_{ll'} = \frac{2e^2}{\hbar}\sum_{jj'}\int\frac{d\mathbf{k}_\parallel}{(2\pi)^2}\left(f_{j',\mathbf{k}_\parallel-s\mathbf{Q}} - f_{j,\mathbf{k}_\parallel}\right) \times \frac{s^*_{l,jj'}s_{l',jj'}}{s\omega + {\rm i}\gamma - \left(\varepsilon_{j,\mathbf{k}_\parallel} - \varepsilon_{j',\mathbf{k}_\parallel-s\mathbf{Q}}\right)}. \tag{S33}$$

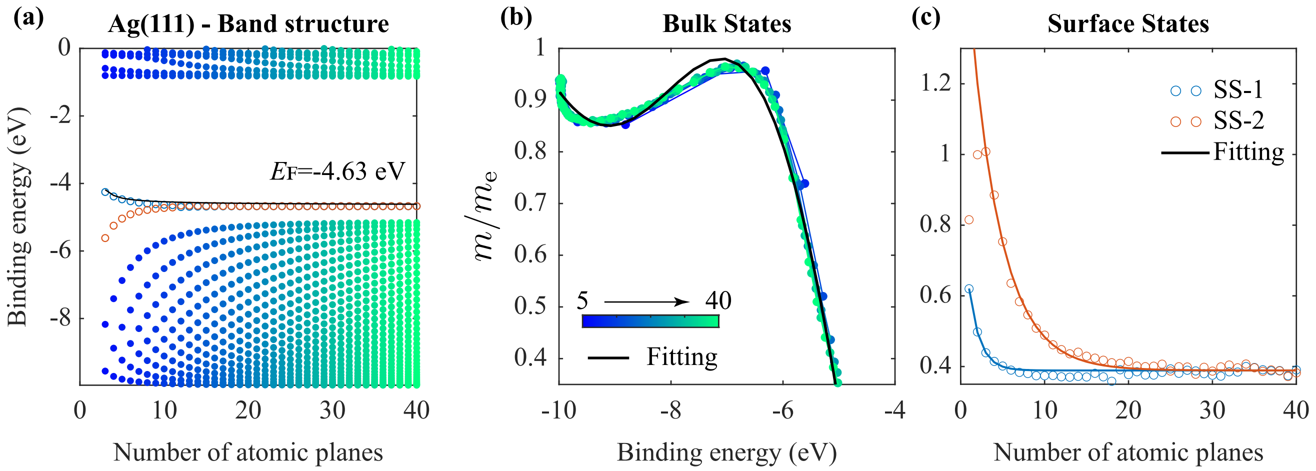

FIG. S1: **Characterization of electronic states in Ag(111) thin films.** (a) We represent the binding energies at the Γ point ($\mathbf{k}_\parallel = 0$, color-coded dots) as calculated from the effective 1D model potential as a function of the number of (111) atomic planes. Energies are referred to the vacuum level, bulk states are indicated by the thickness-dependent color scale in panel (b), surface states by light blue and red circles, and the Fermi energy by a solid black curve. (b) In-plane effective mass calculated from first principles for the bulk states below the projected gap as a function of the binding energy shown in (a), along with the fitting in Eq. (S40). (c) Variation in the surface state binding energy as a function of the number of (111) atomic planes in the film, along with the fitting in Eq. (S41).

We identify the $\chi^{(0,s)}$ as the noninteracting susceptibility in the random-phase approximation (RPA) evaluated at harmonic index s.

In practice, the nonlinear potential at order n and harmonic s is computed self-consistently by first constructing $\eta^{(n,s)}$ from lower-order terms by means of Eqs. (S24) and (S32). Following a standard procedure in the context of RPA calculations, we obtain the induced potential by expressing Eq. (S31) in matrix form and multiplying by $v^{(s)}$ to isolate

$$\phi^{(n,s)} = \left[1 - v^{(s)} \cdot \chi^{(s)}\right]^{-1} \cdot \beta^{(n,s)}, \tag{S34}$$

where the dot denotes matrix multiplication and we have introduced the Coulomb interaction in the sine basis

$$v_{ll'}^{(s)} = \int dz \int dz' s_l(z) s_{l'}(z') v^{(s)}(z, z'), \tag{S35}$$

as well as the quantity

$$\beta_l^{(n,s)} = \left(\phi_l^{\rm ext} + \phi_{{\rm b},l}^{\rm ind}\right) \delta_{n,1} \left(\delta_{s,-1} + \delta_{s,1}\right) + \sum_{l'} v_{ll'}^{(s)} \eta_{l'}^{(n,s)}, \tag{S36}$$

which acts as a source potential.

S3. ELECTRONIC STRUCTURE OF CRYSTALLINE METAL FILMS

The theoretical formalism presented thus far to describe the self-consistent-field optical response of thin films is independent of the confinement potential $V(z)$ appearing in Eq. (S1). In what follows, we elaborate on the methods employed to treat electrons in crystalline metal films incorporating details of atomic corrugation in the out-of-plane direction.

A. Atomic layer potential

To describe a silver crystalline noble metal film of thickness d, we employ the so-called atomic layer potential introduced in Ref. [1],

$$V(z) = \begin{cases} V_{\rm surf}(z - a_s/2), & z < d/2, \\ V_{\rm surf}(d - a_s/2 - z), & z > d/2, \end{cases} \tag{S37}$$

where the potentials $V_{\rm surf}$ are those reported in Ref. [2], which capture the electronic structure of noble metal surfaces in a specific crystallographic orientation by treating them as a semi-infinite stack of atomic planes with interatomic layer spacing $a_s = 0.2361$ terminating at $z = 0$:

$$V_{\rm surf}(z) = \begin{cases} \frac{1}{4(z-z_{\rm i})}\left[{\rm e}^{-\lambda(z-z_{\rm i})} - 1\right], & z < z_{\rm i}, \\ A_3\,{\rm e}^{-\alpha(z-z_{\rm t})}, & z_{\rm i} < z < z_{\rm t}, \\ -A_{20} + A_2 \cos(\eta z), & z_{\rm t} < z < 0, \\ A_{10} + A_1 \cos(2\pi z/a_s), & z > 0. \end{cases} \tag{S38}$$

Here, the coefficients $A_1 = 4.30\,$eV and $A_{10} = -9.640\,$eV are chosen to reproduce the width and position of the noted energy gap, respectively; the space between $z = z_{\rm i}$ and $z = 0$ represents the transition from the solid bulk to free-space, where electron spill out takes place; and the parameters $A_2 = 3.8442$ and $\eta = 48.470\,{\rm nm}^{-1}$ determine the positions of the Fermi level and the surface

states relative to the vacuum level. Five of the remaining six parameters are determined by imposing the continuity of the potential and its first derivative, so that $A_{20} = A_2 - A_1 - A_{10}$, $A_3 = -A_{20} + A_2 \cos(\eta z_t)$, $\alpha = \eta A_2 \sin(\eta z_t)/A_3$, $\lambda = 2\alpha$, and $z_i = \alpha^{-1}\log(-\lambda/4A_3) + z_t$, while the intermediate point $z_t = -5\pi/4\eta$ is fixed with respect to the surface atomic layer in such a way that z_i corresponds to the image plane position, which is important for describing image states [2].

The atomic layer potential of Eq. (S37) then describes a film with outermost atomic planes located at $z = a_s/2$ and $z = d - a_s/2$, and is continuous at $z = d/2$ by construction. Obviously, d must be taken to be an integer multiple of the atomic-layer spacing a_s.

B. Characterization of effective masses

In order to determine the band-dependent effective masses m_j for a thin metal film comprised of a specific number of atomic planes, we examine the energy dispersion bands $E_j(\mathbf{k}_\parallel)$ extracted from *ab initio* calculations (see below). The optical response of the metal is mainly determined by the behavior of electrons at the Fermi level E_F, and consequently, we compute the effective mass at the Fermi wave vector $k = k_F$ by means of a parabolic fit according to

$$\frac{1}{m_j} = \frac{1}{\hbar^2} \frac{\partial^2 E_j(\mathbf{k}_\parallel)}{\partial {k_\parallel}^2}\bigg|_{k_\parallel = k_F}, \qquad \text{(S39)}$$

where the second derivative is calculated numerically from the generated bands. Figure S1a shows the electronic binding energies $\hbar\varepsilon_j^\perp$ for Ag(111) films with respect to the vacuum level as a function of the number of (111) atomic planes, as obtained from the diagonalization of the Hamiltonian in Eq. (S3) when using the atomic layer potential [1, 2]. Here, electronic states are represented by color-coded dots, with bulk states following the thickness-dependent color scale in (b) and surface states (SS) indicated in light blue and red. The binding energies of the latter exhibit a different evolution with thickness that we attribute to the concentration of their wave functions in the vicinity of the metal-dielectric interface. In films consisting of just a few atomic layers, the surface states undergo strong hybridization and energy splitting that is substantially reduced as the film thickness increases to $\gtrsim 20$ atomic planes, with the energy increasingly approaching the semi-infinite surface value of 0.026 meV below the Fermi energy, indicated by the solid black line.

The effective masses computed for each of the considered thicknesses in Figure S1b, are observed to converge towards a particular shape as a function of their binding energies determined in Figure S1a. In Figure S1b, the effective masses of films with thickness ranging from 5 to 40 Ag(111) atomic planes are presented, showing convergence toward the upper end.

Within the valence band, states closer to the Fermi level exhibit a linear variation of the effective mass, in agreement with previous experiments [3]. However, the variation in effective mass for states with energies $\lesssim -6$ eV deviates from the linear trend, motivating the use of density functional theory in the QM calculations with fitted masses. For an arbitrary number of atomic planes, we find a polynomial fit of the effective mass as a function of the number of atomic planes N to exhibit extreme sensitivity to the precision of the fitting coefficients, whereas a more robust result is obtained by using the functional form

$$\frac{m_j}{m_e} = A_1 \sin(B_1 \hbar\varepsilon_j^\perp) + A_2 \cos(B_2 \hbar\varepsilon_j^\perp) + C \qquad \text{(S40)}$$

with coefficients $A_1 = 0.457$, $A_2 = -1.442$, $B_1 = 0.831\,\mathrm{eV}^{-1}$, $B_2 = 0.319\,\mathrm{eV}^{-1}$, and $C = -0.113$, and with binding energies $\hbar\varepsilon_j^\perp$ computed by diagonalizing the Hamiltonian in Eq. (S3). This expression is plotted as a black solid curve in Figure S1b.

For surface sates, the variation of effective mass as a function of the number of atomic planes is shown in Figure S1c, revealing a decrease in effective mass with increasing number of layers that converges to $m/m_e = 0.39$ for Ag(111) films thicker than 20 layers, in agreement with reported experimental values [4]. In this work, we fit the effective mass variation as a function of the number of atomic planes N as

$$\frac{m_{ss}}{m_e} = a_j e^{-b_j N} + c, \qquad \text{(S41)}$$

with j referring the $j = 1$ or $j = 2$ surface state (SS), for which we have coefficients $a_1 = 0.4996$, $a_2 = 1.391$, $b_1 = 0.7675$, $b_2 = 0.2689$, and $c = 0.39$. We note that the color code of panel Figure S1c coincides with the SS representation in Figure S1a.

C. First-principles description of effective masses

We perform density functional theory (DFT) calculations using the Vienna *ab initio* simulation package (VASP) [5–7] using the generalized gradient approximation (GGA) within the Perdew-Burke-Ernzerhof (PBE) formalism [8] for the electron exchange and correlation potential. The interatomic distances are all fixed at the optimized values for the bulk crystal, since relaxation of the silver–silver interatomic distance does not significantly change the electronic band structure, even in films comprised of only a few ($N \sim 5$) atomic planes. Such effects are more important in the outermost surface layers where the interfaces of the film are located, and it is precisely the atomic layer potential model introduced above that incorporates in a phenomenological way the description of the surface fitted to reproduce experimental band parameters.

The cutoff energy of the plane-wave basis set is set to 600 eV. The convergence criterion for the energy is taken

as 10^{-5} eV and the total force in the unit cell is reduced to a value of less than 10^{-4} eV/Å in the optimization of the bulk interlayer distance. A Γ-centered **k**-point mesh of size 18×18×1 is used for the Brillouin zone sampling. A vacuum space of 10 Å is appended in order to prevent interactions between neighboring layers in the vertical supercell.

[1] A. Rodríguez Echarri, J. D. Cox, and F. J. García de Abajo, Optica **6**, 630 (2019).
[2] E. Chulkov, V. Silkin, and P. Echenique, Surf. Sci. **437**, 330 (1999).
[3] M. A. Mueller and T. M. T.-C. Chiang, Phys. Rev. B **41**, 5214 (1990).
[4] F. Reinert, G. Nicolay, S. Schmidt, D. Ehm, and S. Hüfner, Phys. Rev. B **63**, 115415 (2001).
[5] G. Kresse and J. Furthmüller, Phys. Rev. B **54**, 11169 (1996).
[6] G. Kresse and J. Hafner, Phys. Rev. B **47**, 558 (1993).
[7] G. Kresse and J. Furthmüller, Comput. Mater. Sci. **6**, 15 (1996).
[8] J. P. Perdew, K. Burke, and M. Ernzerhof, Phys. Rev. Lett. **77**, 3865 (1996).
